# Where are we now: assessing the price, availability and affordability of essential medicines in Delhi as India plans free medicine for all

**DOI:** 10.1186/1472-6963-13-285

**Published:** 2013-07-25

**Authors:** Anita Kotwani

**Affiliations:** 1 Department of Pharmacology, V. P. Chest Institute, University of Delhi, Delhi 110007, India

**Keywords:** Access, Essential medicines, Generic medicines, Medicine price, Medicine availability, Affordability, Public sector, Private retail pharmacies, Procurement price

## Abstract

**Background:**

Inequitable access to medicines is a major weakness in the Indian health care system. Baseline data needed to develop effective public health policy and provide equitable access to essential medicines. The present survey was conducted to investigate the price, availability, and affordability of fifty essential medicines in the public and private sector in Delhi, India using standardized WHO/HAI methodology.

**Methods:**

Data on procurement price and availability was collected (July-October 2011) from three public healthcare providers: the federal (central) government, state government and Municipal Corporation of Delhi (MCD). Data on price and availability of medicines was collected from private retail and chain pharmacies of a leading corporate house. Prices were compared to an international reference price (expressed as median price ratio-MPR).

**Results:**

The procurement price of surveyed medicines was 0.53-0.82 times the international reference price-IRP. However, the overall mean availability of surveyed medicines in facilities under state government and MCD was 41.3% and 23.2%, respectively. The overall mean availability of medicines in three tertiary care facilities operated by the federal government was 49.3%. Availability of generic medicines was much higher in the private sector. Off-patented medicines, like diazepam, diclofenac, and doxycycline had the highest MPRs. The price ratio between procurement and retail was as high as 28 (range 11–28) for certain medicines. Seven-day treatment with a popular brand of amoxicillin+clavulanic acid or one inhaler each of budesonide and salbutamol cost 2.3 and 1.4 days’ wages for the lowest paid government worker. A majority of India’s population cannot afford these prices.

**Conclusions:**

This study revealed that procurement prices of surveyed medicines were reasonable in comparison to IRP. However, variation in procurement prices of certain medicines by different public procurement agencies was noted. Availability of medicines was very poor in public sector facilities, which are the primary source of free medicines for a majority of India’s low-income population. Availability of medicines is better in private retail pharmacies but affordability remains a big challenge for a majority of the population. These data have significant policy implications that could help in amending policies to increase the access to essential medicines for India’s population.

## Background

Access to healthcare is a fundamental human right, safeguarded internationally and recognized by governments throughout the world [1,2]. In October 2010, the Planning Commission of India constituted the High Level Expert Group (HLEG) on Universal Health Coverage (UHC) with the mandate of developing a framework to provide easily accessible and affordable health care for all Indians [3]. As a first step, the federal government instituted a policy to provide free medicines for all persons attending a government health facility and the first phase was to start by October 2012 [4]. This government initiative is a step in the right direction to provide access to affordable essential medicines [5] and is in line with fulfilling the United Nations Millennium Development Goals [6]. Unfortunately, however, the federal government has already delayed implementation of this policy.

Out-of-pocket payments account for up to 80% of health financing in India [7]. Additionally, 70% of health spending on outpatient treatment goes towards purchasing medicines [7]. Hence, the price of essential medicines really does matter – not only to patients, but also to the government that has embraced the responsibility to provide healthcare to their citizens.

This article describes the detailed reporting of procurement prices and availability of a basket of essential medicines in three major public healthcare providers and the prices, availability and affordability of these medicines for patients in the private sector in National Capital Territory (NCT) of Delhi, India. This survey utilized the standardized and validated methodology of World Health Organization (WHO) and Health Action International (WHO/HAI) [8,9]. Data was collected from July-October 2011 in NCT, Delhi.

## Methods

### Background

Delhi, the capital of India is known as the National Capital Territory of Delhi (NCT Delhi). NCT Delhi has its own elected government but technically it is a federally (centrally) administered state. The NCT Delhi is divided into eight administrative districts.

Three main public sector health care providers in NCT Delhi are: the Central (federal) Government under the Ministry of Health and Family Welfare (MoH & FW), the Directorate of Health Services (DHS) in the Government of NCT (GNCT) Delhi and another public sector provider in Delhi city – the Municipal Corporation of Delhi (MCD). The central government has 3 tertiary care hospitals, 1 children’s hospital, and approximately 90 dispensaries (primary healthcare). The Central Government dispensaries serve only central government employees (current and retired). All citizens benefit from services at the tertiary care hospitals.

At the state level, the Government of NCT Delhi has two tertiary care hospitals, 6 specialist hospitals, 15 secondary care hospitals and approximately 213 dispensaries. The Municipal Corporation of Delhi (MCD) operates 2 tertiary care hospitals, 14 polyclinic/secondary care hospitals and 58 dispensaries. All citizens can visit and avail free services at all government-funded health facilities within NCT Delhi.

Interestingly, all the three healthcare providers have their own procurement system, essential medicine list (EML) or procurement list of medicine and procurement price.

Recently, chain pharmacies have entered the Indian private retail sector. Therefore, both types of retail pharmacies were included in the survey, the traditional private retail pharmacies and the chain pharmacies of a leading corporate house.

### Sampling

Both public and private sector facilities were included in the study. The survey was conducted in all the eight districts of Delhi and five randomly selected facilities were surveyed in each district.

### Public sector

Procurement prices were collected from all the three centralized public sector procurement agencies. The three procurement agencies for each healthcare provider are: Central procurement agency (CPA) for Delhi state government; procurement department of MCD; and Medical Stores Organization (MSO), procurement department of central government. Three tertiary care facilities under the central government (CG) – Lady Hardinge (LH) Medical College and associated hospitals, Ram Manohar Lohia (RML) Hospital, and Safdurjung Hospital (SH) also undertake independent medicine procurements to augment the supply received from their central agency (MSO). RML and SH have a common pooled procurement tender system. Therefore, procurement price data was also collected from RML and from LH in addition to the three centralized procurement agencies.

Medicines prescribed at public facilities are available at no cost, provided they are available at the facility. Therefore, medicine availability was collected from the various public facilities to determine access to medicines. For each district, one secondary care hospital and four primary care centers (dispensaries) were randomly selected. WHO/HAI methodology requires one tertiary care to be included in the survey; therefore, in one of the districts one tertiary care hospital instead of secondary care was selected. Three tertiary care level facilities under the central government were also included. Thus, a total of 83 facilities (40 facilities under GNCT Delhi, 40 under MCD, and 3 for CG) were surveyed.

### Private sector

Private sector facilities were identified by selecting one retail pharmacy outlet in each sector that was in geographic proximity to the nearest public facility. In each district, five retail pharmacies and five retail chain pharmacies were included. Thus a total of 80 facilities were surveyed.

### Medicines surveyed

A total of 50 medicines were surveyed. WHO/HAI methodology has identified 30 core medicines – 14 essential medicines for global burden of disease and 16 specific to South East Asia. Apart from these core medicines a supplementary list of medicines (20) were added; the names, strength and dosage forms are detailed in Table 1. The supplementary list included 17 commonly used antibiotics based on their inclusion in the Delhi state EML; two inhalers that are commonly used for asthma; and a dispersible zinc sulphate tablet, which is recommended by the WHO for acute diarrhoea in children.

**Table 1:** List of medicines surveyed

**A. List of global and WHO-SEARO regional list (30)**			
Amitriptyline	25 mg	cap/tab	Global
Amlodipine	5 mg	cap/tab	Regional
Amoxicillin	500 mg	cap/tab	Global
Amoxicillin suspension	25 mg/ml	milliliter	Regional
Atenolol	50 mg	cap/tab	Global
Atorvastatin	10 mg	cap/tab	Regional
Beclomethasone inhaler	200 mcg/dose	dose	Regional
Captopril	25 mg	cap/tab	Global
Ceftriaxone injection	1 g/vial	vial	Global
Ciprofloxacin	500 mg	cap/tab	Global
Clotrimazole topical cream	1%	gram	Regional
Co-trimoxazole suspension	8+40 mg/ml	milliliter	Global
Diazepam	5 mg	cap/tab	Global
Diclofenac	50 mg	cap/tab	Global
Diethylcarbamazine citrate	50 mg	cap/tab	Regional
Doxycycline	100 mg	cap/tab	Regional
Enalapril	5 mg	cap/tab	Regional
Fluoxetine	20 mg	cap/tab	Regional
Gentamicin eye drops	0.30%	milliliter	Regional
Glibenclamide	5 mg	cap/tab	Global
Gliclazide	80 mg	cap/tab	Regional
Ibuprofen	400 mg	cap/tab	Regional
Metformin	500 mg	cap/tab	Regional
Metronidazole	400 mg	cap/tab	Regional
Omeprazole	20 mg	cap/tab	Global
Paracetamol suspension	24 mg/ml	milliliter	Global
Phenytoin	100 mg	cap/tab	Regional
Ranitidine	150 mg	cap/tab	Regional
Salbutamol inhaler	100 mcg/dose	dose	Global
Simvastatin	20 mg	cap/tab	Global
**B. Supplementary list of medicines (20)**			
Amoxicillin+clavulanic acid	500 mg+125 mg	cap/tab	Supplementary
Amoxicillin 250	250 mg	Tab/cap	Supplementary
Amoxicillin+clavulanic acid Syrup	200 mg+28.5 mg/5 ml	milliliter	Supplementary
Ampicillin Suspension	125 mg/5 ml	milliliter	Supplementary
Azithromycin	500 mg	Tab/cap	Supplementary
Benzathine Penicillin Powder	2.4 MU/vial	inj	Supplementary
Budesonide inhaler	100 mcg/dose	dose	Supplementary
Budesonide+Formoterol inhaler	100 mcg+6 mcg/dose	dose	Supplementary
Cefixime	200 mg	Tab/cap	Supplementary
Cefuroxime axetil	250 mg	Tab/cap	Supplementary
Cefuroxime Suspension	125 mg/5 ml	milliliter	Supplementary
Cephalexin	500 mg	Tab/cap	Supplementary
Cephalexin Syrup	250 mg/5 ml	milliliter	Supplementary
Erythromycin powder for suspension	125 mg/5 ml	milliliter	Supplementary
Erythromycin stearate	250 mg	Tab/cap	Supplementary
Gentamicin injection	40 mg/ml	inj	Supplementary
Norfloxacin	400 mg	Tab/cap	Supplementary
Ofloxacin	200 mg	Tab/cap	Supplementary
Roxithromycin	50 mg	Tab/cap	Supplementary
Zinc sulphate dispersible	20 mg	Tab/cap	Supplementary

WHO/HAI methodology suggests that data for each medicine should be collected for both the originator brand, defined as the product that was first authorised for worldwide marketing, and for the lowest-priced generic equivalent found at each facility. Until 2005, however, product patent protection was not applicable in India and pharmaceutical companies could manufacture medicines that were still under patent in other parts of the world, and this allowed the Indian generic industry to flourish. Consequently, the Indian market has generic versions of all common medications. Nevertheless, medicine manufacturers in India want to generate brand name recognition for their product. Therefore, all products have a brand (trade) name [10,11].

Medicines in India are known as ‘branded’ and ‘branded-generics’. Branded medicines are manufactured by reputable Indian manufacturers and multinational pharmaceutical companies. Branded medicines tend to be more expensive than branded-generics, but are prescribed and sold more often. Financial incentives may spur doctors to prescribe branded medicines; but, physicians also believe that medicines manufactured by leading companies are more likely to be of better quality. Originators brands (OBs) in India are also pooled with branded medicines (popular brands) and as such do not have any additional recognition as originator brand. Frequently OBs are not available but the same molecules are manufactured by other companies with different trade names that are also recognized as ‘branded’ products. Therefore, for this survey, in addition to collecting data for OB and lowest-priced generic (LPG) a third category, highest-priced generic (HPG) data was collected for a list of 30 core medicines at each private sector facility. For supplementary medicines (20), highest-priced and lowest-priced generics were surveyed at each private facility.

Public sector facilities only have one version of each medicine, the lowest-priced generic (LPG). Therefore, data for price and availability were collected for LPG from public sector facilities.

### Data collection entry and analysis

Trained data collectors visited enrolled facilities and recorded medicine availability and prices using a standardized form. Several validation and data checking steps during and after data collection ensured data quality. Data were double entered in Microsoft Excel Workbook and the auto checker was used to assist in the verification process [8].

The price collected from private sector is the price charged directly to patients by each facility. For public sector facilities, procurement prices were collected from three central agencies - CPA for Delhi state government; procurement department of MCD; and Medical Stores Organization (MSO), procurement department of CG. Procurement prices were also collected from two decentralized sites of CG - RML Hospital (RML/SH) and LH Medical College and associated hospitals. Medicine availability was also assessed at all public facilities.

Each sector’s data were analysed separately. Medicine availability is reported as the percent availability of an individual medicine at the surveyed outlets on the day of survey. Mean (average) percent availability across the basket of medicines surveyed is also reported. To facilitate international comparisons, medicine prices are expressed as median price ratio (MPR) [12]. The MPR is the local median unit price of a medicine in comparison with the median unit price found in the Management Sciences for Health (MSH) Price Indicator Guide, 2010 (MSH 2010), i.e. the international reference price [13]. MSH international reference prices represent actual procurement prices for medicines offered to low-income and middle-income countries by non-profit suppliers and international tender prices. MPR of 1 or less is taken as efficient procurement in the public sector, while below 2.5 is considered efficient for the private sector [14]; availability less than 30% is considered very low and greater than 80% is considered high [14]. Data was analyzed using Microsoft Excel.

The WHO/HAI methodology also measures the affordability, which is defined as the number of days an unskilled government worker must work to purchase a standard treatment regimen for common clinical conditions. Treatment affordability is calculated using the cost of a one month treatment for chronic diseases or the cost of the course of therapy for acute diseases.

### Ethical approval

Ethical approval of the study was obtained from Vallabhbhai Patel Chest Institute, University of Delhi, India. Permission for data collection was obtained from Health Department, Directorate Health Services (DHS) of Government of NCT Delhi, Municipal Corporation of Delhi, and from Ministry of Health & Family Welfare, Government of India.

## Results

### Public sector

#### Procurement price

Procurement price data collected for surveyed medicines from three centralized procurement agencies and two decentralized sites are shown in Table 2. Number of medicines being procured out of 50 surveyed medicines, median MPR with inter-quartile ranges for the procured medicines, and minimum and maximum MPR of medicines for each procurement agency are mentioned in the Table 2.

**Table 2:** Summary of median price ratios (MPRs) in various public sectors in Delhi

**Procurement agency (number of**	**CPA (n =37)**	**MCD (n =31)**	**MSO (n =12)**	**LH (n =32)**	**RML/SH (n =27)**
**medicines purchased out of 50 medicines)**					
**Median MPR**	0.61	0.59	0.53	0.82	0.69
**25%ile MPR**	0.42	0.37	0.30	0.61	0.48
**75%ile MPR**	0.76	0.76	0.64	1.10	0.87
**Minimum MPR**	0.07 (Amlodipine)	0.07 (Amlodipine)	0.10 (Amlodipine)	0.34 (Ranitidine)	0.08 (Amlodipine)
**Maximum MPR**	1.22 (Amoxicillin 500 mg)	1.22 (Amoxicillin 500 mg)	1.03 (Erythromycin tab)	6.38 (Fluoxetine tab)	1.48 (Erythromycin tab)

CPA – procurement agency for Delhi state government, MCD – Municipal Corporation of Delhi, MSO – Procurement agency for Central government, LH – a tertiary care facility of central government procurement, RML – tertiary care facility under central government that does procurement for two hospitals.

Note: MPR of Amlodipine was 0.42 for LH; MPR of Erythromycin tab was 0.61 for CPA, 0.96 for MCD, and 1.21 for LH; MPR of Fluoxetine was 0.50 for CPA and was not procured by MCD, MSO, RML/SH.

A few medicines, like atenolol, ceftriaxone injection, diazepam, diclofenac, enalapril, erythromycin stearate, fluoxetine, metformin, and phenytoin were found to have substantial variation in procurement price by different agencies. Most often the tertiary care facility carrying out independent procurement had the highest procurement price for these medicines.

#### Availability of surveyed medicines

**Figure 1:**
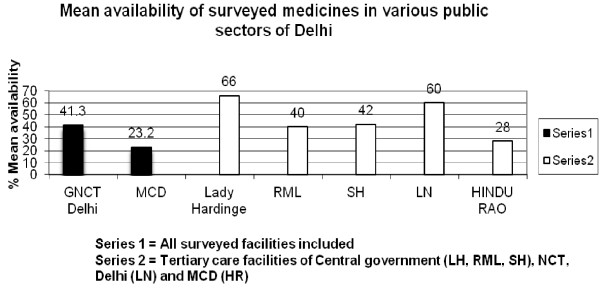
Percent mean availability of surveyed medicines in various public sectors.

The overall mean availability of surveyed medicines in all surveyed facilities under GNCT, Delhi, and MCD was 41.3% and 23.2% (Figure 1). The availability of surveyed medicines in three tertiary care hospitals under CG and one tertiary care hospital each under GNCT, Delhi and MCD was 60.0%, 40.0%, 42.0%, 60.0% and 28.0% (Figure 1).

Government of NCT, Delhi maintains an EML which is used for procurement of medicines at facilities under its purview. Out of 50 medicines surveyed, 40 medicines were on the Delhi state EML at the time of survey. Mean availability for these 40 medicines was 48.8% in Delhi state operated facilities. Other branches in the public sector, MCD and hospitals under Central Government do not have their own EML but a procurement list is prepared by their respective departments. MCD had a procurement price for 33 medicines from the basket of 50 medicines surveyed. The mean availability of these 33 medicines was 34.0%.

Five medicines were not available at any of the facilities surveyed under Delhi state government. In other words, these five medicines had 0% availability: beclomethasone inhaler, budesonide+formoterol inhaler, captopril, gliclazide, and dispersible zinc tablet. All five medicines were not on the EML of Delhi state government. On the other hand, MCD had 15 medicines with 0% availability at the surveyed facilities.

Nine medicines were available in the range of 81-100% at facilities under Delhi state government (Tables 3, 4 and 5) and these were: amlodipine, amoxicillin 250 mg, atenolol, ibuprofen, norfloxacin, omeprazole, paracetamol suspension, ranitidine and salbutamol inhaler. In MCD facilities only three medicines were in this range: amlodipine, atenolol, and omeprazole.

**Table 3:** Availability of surveyed medicines for chronic diseases in various public sector facilities in NCT, Delhi, India

**Medicines**	**GNCT Delhi (n=40)**	**MCD (n=40)**	**CG (LH)**	**CG (RML)**	**CG (SJH)**	**GNCTD (LNH)**	**MCD (HRH)**
		**(Depression Psychoanaleptics)**					
Amitriptyline	10.0%	0.0%	Available	N.A	N.A	N.A	N.A
Fluoxetine	2.5%	0.0%	Available	N.A	Available	N.A	N.A
		**Hypertension**					
Amlodipine	92.5%	90.0%	Available	N.A	Available	Available	Available
Atenolol	85.0%	90.0%	Available	Available	Available	Available	Available
Enalapril	42.5%	47.5%	Available	Available	N.A	Available	N.A
		**Diabetes**					
Glibenclamide	77.5%	42.5%	Available	N.A	N.A	N.A	N.A
Gliclazide	0.0%	35.0%	N.A	N.A	N.A	N.A	N.A
Metformin	60.0%	70.0%	Available	Available	Available	Available	Available
		**Acid related disorder**					
Ranitidine	85.0%	17.5%	Available	Available	Available	Available	N.A
Omeprazole	85.0%	85.0%	Available	N.A	Available	Available	Available
		**Epilepsy**					
Phenytoin	60.0%	15.0%	Available	Available	Available	Available	Available
		**Hyperlipidemia**					
Atorvastatin	27.5%	2.5%	Available	N.A	N.A	Available	Available
Simvastatin	2.5%	0.0%	N.A	N.A	N.A	N.A	N.A
		**Obstructive airways diseases**					
Beclomethasone inhaler	0.0%	0.0%	N.A	N.A	N.A	N.A	N.A
Budesonide inhaler	22.5%	5.0%	Available	N.A	N.A	Available	N.A
Budesonide+Formoterol inhaler	0.0%	0.0%	N.A	N.A	N.A	N.A	N.A
Salbutamol inhaler	85.0%	0.0%	N.A	N.A	N.A	Available	N.A

GNCT Delhi: Government of NCT Delhi; MCD: Municipal Corporation of Delhi; CH: Central government; LH: Lady Hardinge Medical College and Hospital; RML: Ram Manohar Lohia Hospital; SJH: Safdarjung Hospital; LNH: Lok Nayak Hospital; HRH: Hindu Rao Hospital.

Percent availability depicts availability of medicines in all the facilities surveyed for Government of NCT Delhi and for Municipal Corporation of Delhi. Available and Not available (N.A) is used for availability of the medicine for only one tertiary care facility.

Availability depicted as 0.0% indicates the medicine was not available in any of the 40 facilities surveyed.

Availability depicted as N.A indicates that the particular medicine is not available in the mentioned tertiary care facility on the day of the survey.

**Table 4:** Availability of surveyed medicines for acute diseases in various public sector facilities in NCT, Delhi, India

**Medicines**	**GNCT Delhi (n=40)**	**MCD (n=40)**	**CG (LH)**	**CG (RML)**	**CG (SJH)**	**GNCTD (LNH)**	**MCD (HRH)**
	**Antibacterial**						
Amoxicillin+Clavulanic acid	27.5%	52.5%	Available	N.A	Available	Available	Available
Amoxicillin 500	70.0%	20.0%	Available	N.A	N.A	Available	Available
Azithromycin	2.5%	2.5%	Available	N.A	N.A	N.A	N.A
Benzathine Penicillin Powder	2.5%	0.0%	N.A	Available	Available	Available	N.A
Cefixime	20.0%	27.5%	N.A	N.A	N.A	N.A	Available
Ceftriaxone injection	12.5%	0.0%	Available	Available	Available	Available	N.A
Cefuroxime axetil	50.0%	47.5%	N.A	N.A	N.A	Available	N.A
Cephalexin	47.5%	20.0%	N.A	Available	N.A	N.A	N.A
Ciprofloxacin	50.0%	72.5%	Available	Available	Available	Available	Available
Doxycycline	52.5%	70.0%	Available	N.A	N.A	N.A	Available
Erythromycin Stearate	47.5%	0.0%	Available	Available	Available	Available	N.A
Gentamicin injection	10.0%	0.0%	Available	Available	Available	Available	N.A
Norfloxacin	82.5%	2.5%	Available	Available	Available	N.A	N.A
Ofloxacin	45.0%	47.5%	Available	Available	N.A	Available	Available
Roxithromycin	47.5%	7.5%	N.A	N.A	N.A	Available	N.A
	**Antifungal for topical use**						
Clotrimazole topical cream	62.5%	2.5%	N.A	N.A	Available	Available	N.A
	**Psycholeptic**						
Diazepam	10.0%	5.0%	Available	Available	Available	N.A	N.A
	**Antifilarial**						
Diethylcarbamazine citrate	2.5%	0.0%	N.A	N.A	N.A	N.A	N.A
	**Anti-inflammatory/antirheumatic**						
Diclofenac	35.0%	7.5%	Available	Available	N.A	Available	N.A
Ibuprofen	92.5%	15.0%	Available	Available	Available	Available	N.A
	**Antibiotic for ophthalmic use**						
Gentamicin eye drops	62.5%	12.5%	Available	N.A	Available	Available	N.A
	**Amoebia and other protozoal infections**						
Metronidazole	57.5%	32.5%	Available	N.A	Available	N.A	N.A

GNCT Delhi: Government of NCT Delhi; MCD: Municipal Corporation of Delhi; CH: Central government; LH: Lady Hardinge Medical College and Hospital; RML: Ram Manohar Lohia Hospital; SJH: Safdarjung Hospital; LNH: Lok Nayak Hospital; HRH: Hindu Rao Hospital.

Percent availability depicts availability of medicines in all the facilities surveyed for Government of NCT Delhi and for Municipal Corporation of Delhi. Available and Not available (N.A) is used for availability of the medicine for only one tertiary care facility.

**Table 5:** Availability of surveyed paediatric medicines in various public sector facilities in NCT, Delhi, India

**Medicines**	**GNCT, Delhi (n=40)**	**MCD (n=40)**	**CG (LH)**	**CG (RML)**	**CG (SJH)**	**GNCTD (LNH)**	**MCD (HRH)**
Amoxicillin suspension	70.0%	5.0%	Available	N.A	N.A	Available	N.A
Amoxicillin 250	82.5%	32.5%	Available	Available	Available	Available	N.A
Amoxicillin+Clavulanic acid Syrup	30.0%	60.0%	N.A	N.A	N.A	Available	Available
Ampicillin Suspension	22.5%	2.5%	N.A	N.A	N.A	Available	N.A
Cefuroxime Suspension	5.0%	0.0%	N.A	N.A	N.A	N.A	N.A
Cephalexin Syrup	47.5%	7.5%	Available	N.A	N.A	Available	N.A
Co-trimoxazole suspension	35.0%	30.0%	Available	Available	N.A	N.A	N.A
Paracetamol suspension	97.5%	75.0%	Available	Available	Available	Available	Available
Zinc sulphate dispersible	0.0%	0.0%	N.A	N.A	N.A	N.A	N.A

GNCT Delhi: Government of NCT Delhi; MCD: Municipal Corporation of Delhi; CH: Central government; LH: Lady Hardinge Medical College and Hospital; RML: Ram Manohar Lohia Hospital; SJH: Safdarjung Hospital; LNH: Lok Nayak Hospital; HRH: Hindu Rao Hospital.

Percent availability depicts availability of medicines in all the facilities surveyed for Government of NCT Delhi and for Municipal Corporation of Delhi. Available and Not available (N.A) is used for availability of the medicine for only one tertiary care facility.

#### Availability of surveyed medicines for chronic and acute diseases

Table 3 and Table 4 show medicine data categorized by use in chronic and acute diseases. Availability of medicines for treatment of depression, hyperlipidemia, and obstructive airways diseases was very poor in all facilities. Availability of medicines for acute conditions was not optimum in the public sector either. In general, availability of medicines was poorer in MCD facilities compared to Delhi state government facilities.

#### Availability of surveyed paediatric medicines

Paracetamol suspension, a core medicine, was available at almost all public outlets but the availability was 75% at MCD facilities (Table 5). Overall availability of two antibacterial core medicines, amoxicillin suspension and co-trimoxazole suspension was poor in the public sector. Zinc sulphate dispersible tablets were not available in any of the facilities.

### Private sector

#### Price-to-patient

The median MPR for all versions, inter-percentile range, minimum and maximum MPR found at retail pharmacies and retail chain pharmacies are shown in Table 6. Findings were similar at both retail pharmacies and retail chain pharmacies.

**Table 6:** Summary of medicine prices in the private sector

	**Private pharmacies**			**Chain pharmacies**		
	**Brand (n =16)**	**Highest priced (n =28)**	**Lowest priced (n =43)**	**Brand (n =17)**	**Highest priced (n =29)**	**Lowest priced (n= 43)**
Median MPR	4.71	5.38	2.83	4.41	4.79	3.12
25%ile MPR	2.59	2.76	1.74	2.21	2.53	1,79
75%ile MPR	7.42	6.84	5.20	7.18	6.78	4.94
Minimum MPR	0.57	0.57	0.56	0.57	0.57	0.56
Maximum MPR	16.51	9.30	9.73	16.51	11.26	9.73

Note: Minimum MPR was for ranitidine for all three versions. Maximum MPR for brand medicine was diclofenac, for highest-priced was for doxycycline, and for the lowest-priced generic was for diazepam. The results were same for chain pharmacies.

#### Price variations in public and private sector

Median MPR of lowest-priced generic (LPG) in the private sector for diazepam, amlodipine, atenolol, enalapril, diclofenac, and glibenclamide was 28, 23, 22, 16, 14 and 11 times higher while two paediatric antibiotics, cefuroxime suspension and cephalexin syrup were seven times more costly than the MPR of these medicines in the public sector. On the other hand, budesonide inhaler, co-trimoxazole suspension, erythromycin, gentamicin injection, and salbutamol inhaler did not show much price variation from one sector to another.

#### Price variation between brand, highest-priced generic and lowest-priced generic

**Figure 2:**
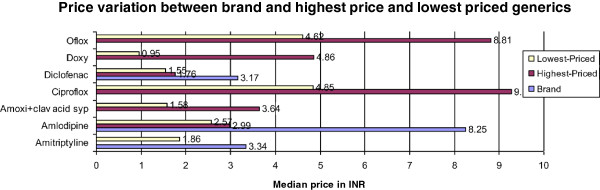
Price variation for a few medicines in brand, highest and lowest-priced generics available at private sector.

**Figure 3:**
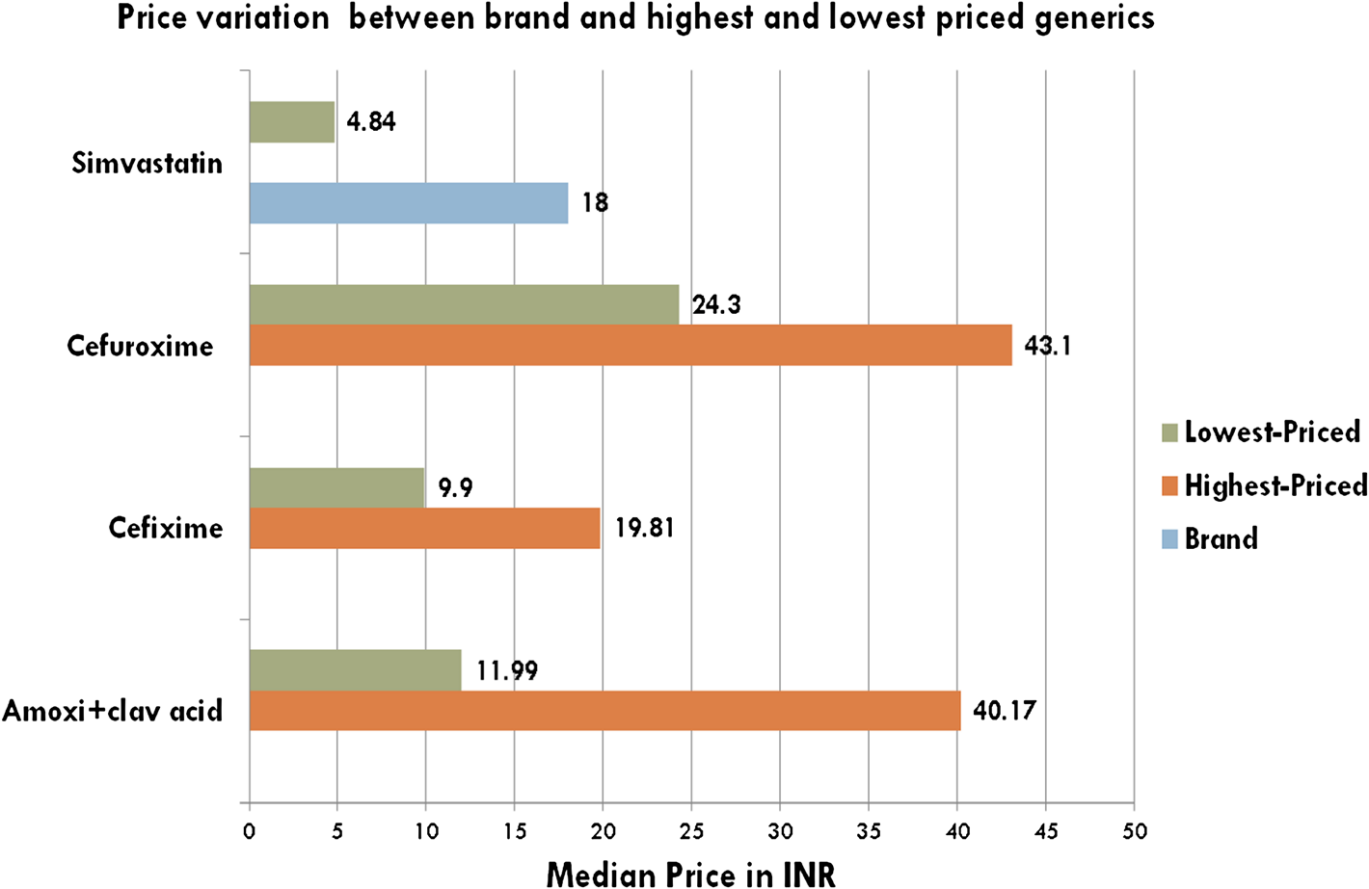
**Price variation for selected medicines for brand, highest and lowest-priced generics available at private sector.** Note: INR = Indian Rupees.

For certain medicines tremendous price variation was observed for brand, highest-priced and lowest-priced generic available at the surveyed facilities (Figures 2 and 3).

#### Overall availability

The percent availability of surveyed medicines in three different categories at private retail pharmacies and chain pharmacies is shown in Table 7. Originator brand was surveyed and identified for 30 core medicines. If only one version of the medicine (besides the originator brand) was available then it was considered to be the lowest-priced generic available.

**Table 7:** Availability of medicine surveyed in different versions in private sector

		**OB**		**HPG**		**LPG**	
**Medicine name, strength**	**Medicine list**	Retail*	**Chain#**	Retail*	**Chain#**	Retail*	**Chain#**
**and dosage form**		Retail* (n = 40)	**(n = 40)**	**(n = 40)**	**(n = 40)**	**(n = 40)**	**(n = 40)**
Amitriptyline 25 mg cap/tab	Global	65.0%	80.0%	2.5%	0.0%	12.5%	12.5%
Amlodipine 5 mg cap/tab	Regional	52.5%	60.0%	82.5%	90.0%	95.0%	100.0%
Amoxicillin+clavulanic acid 500 mg+125 mg cap/tab	Supplementary	NS**	NS	95.0%	100.0%	100.0%	100.0%
Amoxicillin 500 mg cap/tab	Global	0.0%	0.0%	95.0%	92.5%	97.5%	100.0%
Amoxicillin 250 mf cap/tab	Supplementary	NS	NS	72.5%	97.5%	92.5%	100.0%
Amoxicillin suspension 25 mg/ml	Regional	0.0%	0.0%	57.5%	70.0%	95.0%	92.5%
Amoxicillin+clavulanic acid Syrup 200 mg+28.5 mg/5 ml	Supplementary	NS	NS	62.5%	90.0%	92.5%	92.5%
Ampicillin Suspension 125 mg/5 ml	Supplementary	NS	NS	2.5%	0.0%	2.5%	0.0%
Atenolol 50 mg cap/tab	Global	45.0%	45.0%	67.5%	92.5%	97.5%	100.0%
Atorvastatin 10 mg cap/tab	Regional	12.5%	30.0%	87.5%	85.0%	97.5%	95.0%
Azithromycin 500 mg cap/tab	Supplementary	NS	NS	95.0%	97.5%	100.0%	100.0%
Beclomethasone inhaler 250 mcg/dose	Regional	5.0%	0.0%	0.0%	0.0%	35.0%	37.5%
Benzathine Penicillin Powder 2.4 MU/vial	Supplementary	NS	NS	0.0%	0.0%	0.0%	0.0%
Budesonide inhaler 100 mcg/dose	Supplementary	NS	NS	7.5%	10.0%	62.5%	82.5%
Budesonide+Formoterol inhaler 100 mcg+6mcg/dose	Supplementary	NS	NS	0.0%	0.0%	42.5%	27.5%
Captopril 25 mg cap/tab	Global	0.0%	0.0%	0.0%	0.0%	10.0%	10.0%
Cefixime 200 mg cap/tab	Supplementary	NS	NS	80.0%	95.0%	97.5%	100.0%
Ceftriaxone injection 1 g/vial	Global	0.0%	0.0%	2.5%	5.0%	47.5%	42.5%
Cefuroxime axetil 250 mg cap/tab	Supplementary	NS	NS	55.0%	60.0%	90.0%	100.0%
Cefuroxime Suspension 125 mg/5 ml	Supplementary	NS	NS	2.5%	0.0%	55.0%	50.0%
Cephalexin 500 mg cap/tab	Supplementary	NS	NS	32.5%	30.0%	65.0%	80.0%
Cephalexin Syrup 250 mg/5 ml	Supplementary	NS	NS	22.5%	22.5%	50.0%	40.0%
Ciprofloxacin 500 mg cap/tab	Global	0.0%	0.0%	85.0%	92.5%	100.0%	100.0%
Clotrimazole topical cream 1%	Regional	50.0%	55.0%	10.0%	17.5%	50.0%	67.5%
Co-trimoxazole suspension 8+40 mg/ml	Global	5.0%	10.0%	0.0%	0.0%	77.5%	67.5%
Diazepam 5 mg cap/tab	Global	35.0%	52.5%	5.0%	2.5%	37.5%	57.5%
Diclofenac 50 mg cap/tab	Global	97.5%	97.5%	10.0%	12.5%	65.0%	82.5%
Diethylcarbamazine citrate 50 mg cap/tab	Regional	12.5%	10.0%	0.0%	0.0%	10.0%	10.0%
Doxycycline 100 mg cap/tab	Regional	0.0%	0.0%	55.0%	60.0%	97.5%	95.0%
Enalapril 5 mg cap/tab	Regional	0.0%	0.0%	42.5%	32.5%	92.5%	97.5%
Erythromycin powder for suspension 125 mg/5 ml	Supplementary	NS	NS	15.0%	15.0%	55.0%	55.0%
Erythromycin Stearate 250 mg cap/tab	Supplementary	NS	NS	35.0%	25.0%	82.5%	85.0%
Fluoxetine 20 mg cap/tab	Regional	0.0%	0.0%	42.5%	62.5%	80.0%	92.5%
Gentamicin eye drops 0.3%	Regional	5.0%	2.5%	2.5%	0.0%	65.0%	50.0%
Gentamicin injection 40 mg/ml	Supplementary	NS	NS	15.0%	17.5%	57.5%	50.0%
Glibenclamide 5 mg cap/tab	Global	65.0%	72.5%	0.0%	0.0%	5.0%	7.5%
Gliclazide 80 mg cap/tab	Regional	0.0%	2.5%	55.0%	67.5%	95.0%	97.5%
Ibuprofen 400 mg cap/tab	Regional	95.0%	82.5%	0.0%	0.0%	45.0%	40.0%
Metformin 500 mg cap/tab	Regional	0.0%	0.0%	82.5%	80.0%	97.5%	95.0%
Metronidazole 400 mg cap/tab	Regional	45.0%	52.5%	2.5%	5.0%	92.5%	95.0%
Norfloxacin 400 mg cap/tab	Supplementary	NS	NS	5.0%	0.0%	95.0%	97.5%
Ofloxacin 200 mg cap/tab	Supplementary	NS	NS	92.5%	97.5%	97.5%	100.0%
Omeprazole 20 mg cap/tab	Global	0.0%	0.0%	87.5%	95.0%	100.0%	100.0%
Paracetamol suspension 24 mg/ml	Global	62.5%	65.0%	20.0%	17.5%	90.0%	97.5%
Phenytoin 100 mg cap/tab	Regional	37.5%	45.0%	32.5%	40.0%	90.0%	95.0%
Ranitidine 150 mg cap/tab	Regional	95.0%	85.0%	82.5%	87.5%	97.5%	95.0%
Roxithromycin 50 mg cap/tab	Supplementary	NS	NS	17.5%	25.0%	92.5%	97.5%
Salbutamol inhaler 100 mcg/dose	Global	52.5%	45.0%	7.5%	7.5%	92.5%	95.0%
Simvastatin 20 mg cap/tab	Global	42.5%	42.5%	2.5%	0.0%	40.0%	35.0%
Zinc sulphate dispersible 20 mg cap/tab	Supplementary	NS	NS	0.0%	0.0%	0.0%	2.5%

*Retail indicates the retail pharmacies shops and #Chain indicates retail chain pharmacies.

*OB* – Originator Brand.

*HPG* – Highest priced generic.

*LPG* – Lowest priced generic.

**NS – Not surveyed.

#### Medicines with poor availability

A few medicines, such as benzathine penicillin powder, captopril, diethylcarbamazepine, erythromycin powder for suspension, and zinc dispersible tablet had poor overall availability.

#### Medicines with only one version available

A few medicines were generally available with only one trade name which was usually the most popular brand name. These medicines were: beclomethasone, budesonide, budesonide+formoterol and salbutamol inhalers, ceftriaxone injection, cefuroxime suspension, cephalexin, cephalexin suspension, clotrimazole cream, co-trimoxazole suspension, gentamicin eye drops, gentamicin injection, glibenclamide, metronidazole, norfloxacin, and roxithromycin.

### Affordability

**Figure 4:**
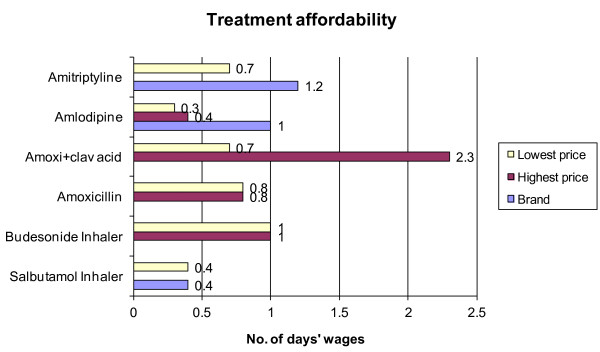
**Treatment affordability for depression, hypertension, acute respiratory infection and asthma treatment in private medicine outlets.** Note: No. of days’ wages of lowest-paid government worker who earns Indian Rupees 247 (USD $5.5) per day.

Affordability was calculated on the basis of the daily wage of an unskilled government worker. The salary of the lowest paid regular government worker was Indian Rupees (INR) 247 or USD $5.5 (2011) per day. The prices of treatments in the private retail shop and chain pharmacies were almost equal. The cost of treatment and affordability of four conditions is shown in Figure 4.

Calculations of affordability included only the medicine price - physician consultation fees and diagnostic tests will likely mean that the total cost to the patient may be considerably higher.

## Discussion

The present study is perhaps the only study that compares the procurement prices and availability of a basket of essential medicine for three public health care providers in a single state of any country. The study also surveyed the medicine price and availability in the private sector, from the traditional retail pharmacy shops and recently introduced chain pharmacies.

Delhi spans a relatively small geographical area as compared to other Indian states. Therefore, a more detailed study that samples a larger percentage of public and private facilities was possible. Since Delhi is the capital of the country, meetings and rapid dissemination of results to regulatory agencies and policy makers is possible and indeed a meeting was conducted for the stakeholders. This article should be useful to government health policymakers in providing a broad picture of the present situation regarding essential medicines and suggesting ways to strengthen the EMLs, procurement and supply chain that will bring benefit to patients.

Despite the strengths, the WHO/HAI methodology has few limitations. First, availability and price are determined for a specific list of survey medicines, and do not account for alternate dosage forms of these medicines or therapeutic alternates. Second, differences in quality across products are not accounted for. Availability data only refer to the day of data collection at each facility and might not indicate average availability of medicines over time. However, since the survey is done in several facilities over a period of time (2–3 months), the data provide a reasonable estimate of the overall situation and are indicative of the real-life situation faced by the patients on a daily basis.

### Public sector

#### Procurement price

All the three central procurement agencies, CPA of Delhi state government, procurement department of MCD, and MSO of central government have two-bid tender system. Technically qualified companies are eligible to place a price-bid and the rate is fixed with the company that has the lowest quoted price. The median MPR of purchased medicine by all the three agencies was less than one, indicating the procurement price was reasonable as compared to international reference price. Six previous surveys conducted simultaneously in 2004 in five states of India using WHO/HAI methodology showed median MPR in the range of 0.27 – 0.48 for core medicines [15]. The median MPR for core medicines procured by the CPA, Delhi state government was found to be 0.48 in this study.

The central government procurement agency, MSO was procuring only 12 medicines out of 50 surveyed medicines therefore all the three tertiary care facilities under central government make their own parallel procurement. The median MPR for these two decentralized procurement was 0.69 and 0.82 whereas for three central procurement agencies was between 0.53 - 0.61. We found variation in procurement price for the same medicine procured by different agencies. Generally, the procurement price of tertiary care facilities conducting independent (decentralized) procurement was much higher than other agencies. These findings confirm that pooled procurement decreases the procurement price [15,16]. It is established that pooled procurement decreases the medicines’ procurement prices and local purchase of medicines done by individual facility cost higher [17].

#### Policy options for improving procurement process

The procurement agencies in a state or country should keep each other advised on their system and the rate list or procurement prices fixed by the respective agencies. A common list of trustworthy companies who supplied the medicines at required intervals could be circulated to each other. Efforts should be made by the central procurement agency of Central Government (MSO) to be more efficient and to procure adequate numbers of medicines for its facilities. The three facilities under central government can prepare a common EML based on essential medicine concept, combine their requirement of medicines, and opt for pooled procurement. Government of NCT Delhi and MCD can have a common list of essential medicines for primary, secondary and tertiary care facilities which can decrease the replication of work. To implement these policies, transparency and accountability within the health department are a pre-requisite [18].

#### Poor availability of surveyed medicines in the public sector

As mentioned earlier WHO and HAI has set a benchmark of 80% for medicine availability as good [14,19] against which we found that medicines for acute, chronic, and for children were suboptimal. Availability of essential medicines was found to be poor in other low- and middle-income countries [20-22]. Availability of two medicines for hypertension, amlodipine and atenolol was good (>80%) in all the public sectors. Availability of medicines for other chronic diseases, like asthma, psychiatric conditions, and hyperlipidemia was very poor. Earlier surveys conducted in other states of India have also shown poor availability of essential medicines in the public sector [15,17]. Budesonide and salbutamol inhalers were on the Delhi state EML but they were not in the procurement list of other two agencies. It is reported earlier that inhalers were not on the procurement list of other Indian states like Haryana, Karnataka, Maharashtra, Tamil Nadu, and West Bengal [23]. Antidepressants agents studied, amitriptyline and fluoxetine were on the Delhi state EML and are also on the National EML 2011 [24] but not on the procurement list of MCD. However, the availability of psychiatric medicines was very poor for Delhi state and for central (federal) government run facilities [25]. Studies from other low-and middle-income countries have shown poor availability of medicines for chronic diseases [26-29]. Dispersible zinc tablet was not procured by any agency though WHO recommends this medicine for treatment of acute diarrhoea in children [30]. The second and third generation antibiotics like cefuroxime, ofloxacin, cefixime, amoxicillin+clavulanic acid were available at primary healthcare facilities.

#### Policy options to improve availability of medicines in the public sector

Low availability of medicines in the public sector could results from factors such as under-budgeting, purchasing medicines not included in the EML, inability to forecast needs accurately, and inefficient purchasing/distribution in the supply chain [18,20]. Various recommendations that can improve availability of essential medicines in the surveyed state are - Government to increase the budget for medicines; prepare standard treatment guidelines (STGs) and EML on the basis of essential medicine concept; separate EML for primary care and hospitals; procurement and distribution of medicines on the basis of EML; efficient transparent and accountable procurement and distribution system; prescriptions according to STGs and EML and; regular monitoring and evaluation of the system [31,32].

### Private sector

Availability of medicines was good in the private sector because of domestic competitive pharmaceutical industry. Findings revealed that availability and price of medicines were similar at traditional retail pharmacies and chain pharmacies.

#### Availability price and affordability

Availability of medicines in the private sector was consistently higher, though the higher prices of medicines could hinder the access. Availability of few medicines was found to be suboptimal; for many medicines, only one version of the product was available that was the costly or branded medicine (popular trade name). Therefore, the patient has no choice but to buy that particular costly branded product. Pharmacists will stock those medicines that are most frequently prescribed. This indicates that doctors tend to prescribe branded medicines. Legally pharmacists are not allowed to substitute between branded and generic medicines. The brand name (trade name) written by a doctor can not be substituted with another. Highest MPRs were found for off-patented medicines, like diazepam, diclofenac and doxycycline which indicates that these medicines are very costly compared to international reference price.

Huge price differences was observed between procurement price and private retail price for certain medicines like diazepam, amlodipine, atenolol, enalapril, diclofenac, glibenclamide, which were 11–28 times more expensive in the retail market than the median public procurement price. Interestingly and unfortunately all these medicines are used for various chronic diseases. A similar finding for diazepam and diclofenac was observed earlier in 2003 and 2004 surveys conducted at six different states of India [15,17]. These findings give a clue that in the supply chain from manufacturer to retailer, one actor or all have huge mark-ups. An earlier study on medicine price components [10] and recent survey on tracking medicine prices in the supply chain in Delhi [33] revealed that the main profit is for the actor who is pushing and responsible for promoting the sale of medicine, i.e., pharmaceutical company for branded products and retailer for the branded-generics.

In India a few essential medicines are under price control, for most of the medicines government does not fix the price. It is believed that free market forces will keep the prices of medicine in-check. Findings from the private sector indicate that the free market competition does not seem to be driving medicine prices as low as possible. Brand loyalty, marketing strategies do not allow ‘real’ competition in free pharmaceutical market [33]. As the free market competition is not working to lower medicine prices, the free market rules need to be modified by the Department of Pharmaceuticals [34] or government must intervene and regulate the prices of essential medicines or provide subsidy for certain formulations that are expensive.

With the lowest daily wage of government worker Indian Rupees 247 (USD $5.5 2011) per day, treatments were not so affordable, e.g., adult respiratory pneumonia if treated with amoxicillin will cost 0.8 days salary and if treated with amoxicillin+clavulanic acid will cost 2.3 days of salary with highest-priced generic. Purchasing one inhaler each of budesonide and salbutamol costs 1.4 days’ wages for the lowest paid government worker. However, a large proportion of India’s population earns less than this. According to World Bank Report, the Gross National Income per capita for the year 2011 in India is $1420/annum or USD $3.89 per day [35]. It is reported that about 320 million people in India are working in unorganized sector and around 300 million people are unemployed [36]. According to Horton and Das World Bank definition of poverty (an income of less than USD $1.25 a day) is more sensitive, embracing 42% of India’s people [37]. Medicines for chronic diseases are often unaffordable as they require lifelong treatment with multiple medications [38]. Further, the need for other mandatory expenditure like food, housing, and other family members living on the salary will change the affordability estimate. Affordability can be severely affected by multiple illnesses in the family or if the earning member is one to fall ill. Therefore, the information on affordability is to be interpreted with caution and should not impact on the potential for taking policy decisions for medicine prices in India.

#### Policy options to improve access to affordable medicines

Awareness programmes targeting various stakeholders like doctors, patients, consumer groups, and the media are needed. From the various policies suggested [31,39,40] for cost containment and promotion of generics that can be implemented in Indian context for promotion of awareness are: involvement of reputed organizations and institutions to participate in the process; involvement of patient associations and consumers group; reliable quality assurance capacity, including demonstration of bioequivalence; encouraging generic prescribing (in Indian context branded-generic prescribing); influencing the patient to ask for prescription of cheaper version of medicines. A number of countries have used the results of recent surveys of medicine prices and availability to inform and guide policies to improve access to medicines [41]. Lebanon is an example where the government implemented regressive margins for importers, wholesalers and retailers; improved transparency by publishing patient prices on the internet and in the Lebanon National Drug Index. In another example, the Government of Tajikistan abolished 20% VAT on medicines. In India we need to have policy actions based on the evidence generated from medicine price surveys to improve access to essential medicines.

Therefore, there is need to collect data on the quality of branded-generics and generics in India which should be widely disseminated and publicised. Awareness programs and workshops for medical students and doctors should be instituted. These workshops should focus on the disparity in price and availability of different brands of the same medicine [42]. The central government may also abolish the 5% VAT, which is borne by patients. Consumer awareness about medicine prices will be useful in bringing down the overall prices of medicines.

## Conclusion

The present survey provides a clear picture of the poor availability of essential medicines in public sector facilities in Delhi, which are the primary source of medicines for poor populations. Therefore, low-income patients are forced to buy medicines from private sector or forego treatment since affordability is a major issue for a large proportion of the Indian population. Findings have given insight to policymakers about the significance of EML for different healthcare levels, magnitude of pooled procurement, importance of regular supply and quantification of medicines for the public sector and significance of prescribing low priced generic version (branded-generic) of medicines in the private sector. The various policy options suggested including the continuous monitoring and evaluation of the system will be helpful in making the government policy of providing ‘free-medicines-to-all’ a reality. Policymakers have a good opportunity to improve the situation by better spending on medicines and more spending on health.

## Abbreviations

CG: Central Government; CPA: Central Procurement Agency; DHS: Directorate of Health Services; EML: Essential medicine list; GNCT Delhi: Government of NCT Delhi; HAI: Health Action International; HLEG: High Level Expert Group; HPG: Highest-priced Generic; INR: Indian Rupees; LH: Lady Hardinge Medical College; LPG: Lowest-priced Generic; MSH: Management Sciences for Health; MPR: Median Price Ratio; MSO: Medical Stores Organization; MoH&FW: Ministry of Health and Family Welfare; MCD: Municipal Corporation of Delhi; NCT: National Capital Territory; OB: Originator Brand; RML Hospital: Ram Manohar Lohia Hospital; SH: Safdurjung Hospital; UHC: Universal Health Coverage; USD: US Dollar; WHO: World Health Organization.

## Competing interests

The author does not have a commercial or other association that might pose a conflict of interest.

## Pre-publication history

The pre-publication history for this paper can be accessed here:

http://www.biomedcentral.com/1472-6963/13/285/prepub
